# *'Diabetology & Metabolic Syndrome*: providing an open access future for diabetes research'

**DOI:** 10.1186/1758-5996-1-1

**Published:** 2009-08-26

**Authors:** Daniel Giannella-Neto, Marília de Brito Gomes

**Affiliations:** 1 Laboratory for Clinical and Experimental Gastroenterology – LIM07, Hospital das Clínicas, University of São Paulo School of Medicine, São Paulo, Brazil; 2 Biomedical Centre, Medical Specialties Division, State University of Rio de Janeiro, Rio de Janeiro, Brazil

## Abstract

*Diabetology & Metabolic Syndrome (D&MS)*, the official journal of the Brazilian Diabetes Society (SBD), is a new open access, peer reviewed journal publishing research on all aspects of the pathophysiology of diabetes and metabolic syndrome. With the many ongoing and upcoming challenges for diabetes diagnosis, treatment and care, a dedicated journal providing unrestricted access for researchers and health care professionals working in the field of diabetes is needed. *Diabetology & Metabolic Syndrome *aims to fulfil this need.

## Editorial

Among the many good reasons to launch a new open access journal devoted to publishing articles in the field of diabetes research is the fact diabetes is seen as a pandemic condition of unprecedented proportions. In 2007 there were an estimated 246 million cases of diabetes worldwide, with the Western Pacific and the European regions having the highest numbers, approximately 67 and 53 million, respectively. The prevalence of diabetes is projected to rise in both developed and developing countries. In the seven regions of the International Diabetes Federation (IDF) the estimated numbers of cases will be 380 million by 2025. In addition, in many populations, diabetes is most common among the elderly but prevalence rates will significantly rise among comparatively young and productive populations in the developing world [1].

Type 2 diabetes mellitus constitutes about 85% to 95% of all diabetes cases in developed countries and accounts for an even higher percentage in developing countries mostly due to increased urbanization, westernization and economic development, which predispose to obesity due to high consumption of industrialized foods and physical inactivity [2]. Moreover, because of the inefficiency and lack of professional and material resources, the public health systems in developing countries will be facing an increasing number of people with poorly controlled diabetes more predisposed to the devastating microvascular and macrovascular consequences of the disease.

In addition to the increasing numbers of diabetes cases, there are many challenges to face in optimising diabetes care. Highlighting this are studies into the factors, which can help in predicting the likelihood of achieving glycaemic control across various regions of the World. The International Diabetes Management Practice Study (IDMPS) concluded that short disease duration and use of few oral antidiabetic drugs were predictive factors for achieving HbA1C <7% in 9,901 T2DM patients recruited by 937 physicians in Eastern Europe, Asia, Latin America, and Africa [3]. In Asia and South America, absence of microvascular complications was an additional predictor. These findings suggest that early diagnosis and prompt initiation of insulin therapy in patients treated with multiple oral antidiabetic drugs may increase the likelihood of attaining glycaemic targets. In Asia and South America, old age was a predictor, which complies with data from the U.S. Diabetes Prevention Program, showing lifestyle modification was more effective in elderly rather than young people, who may be less compliant because of competing priorities. In Asia, lack of obesity and self-adjustment of insulin dosages were predictors, emphasizing the double hit of obesity and βcell insufficiency in Asian populations. Other region-specific factors relevant to self-care (e.g., Self-Monitoring of Blood Glucose and Self-Adjustment of Insulin) and health care systems (e.g., health insurance coverage and access to specialists and diabetes educators) further reflect the multiple challenges the optimization of diabetes care presents [4].

Another important reason for launching the journal is related to the controversies regarding the concept of metabolic syndrome. The term "metabolic syndrome" refers to the clustering of a number of cardiovascular risk factors (obesity, hypertension, dyslipidaemia and hyperglycaemia), believed to be related to insulin resistance. In the eighties Reaven had insulin resistance as the central and hypothetically causal factor tying the syndrome together [5]. The more recent definition by International Diabetes Federation [6] consider obesity or central obesity as the central factor defining the syndrome if at least other elements of dyslipidaemia, hypertension and hyperglycaemia are present, and where insulin resistance is no longer even a part of the syndrome. Despite the fact that an uncountable number of scientific papers on metabolic syndrome have been published over the last ten years, there are controversies concerning the definition of the metabolic syndrome, the diagnostic criteria used to define it, its aetiology, its usefulness and even its existence [7]. In general, however, differences of opinion surrounding the syndrome are minor.

The concept of metabolic syndrome has good points but it also has weak aspects. For example, the syndrome is an attempt to capture both cardiovascular disease and T2DM risks, which are not necessarily the same things [8]. Metabolic syndrome provides a simple diagnostic set for identifying people in high relative risk to develop cardiovascular disease but does not provide a measure of absolute risk. To obtain the absolute risk, it needs to be used with other risk markers such as smoking, high levels of LDL-C and a family history of heart disease to give the 'overall cardiometabolic risk'. The definition of metabolic syndrome needs further refinement and it requires long-term outcome studies to evaluate the various criteria definitively [7].

Many aspects related the origin of metabolic syndrome become cleared by the words of Borch-Johnsen [9] transcribed here:

"Europe and North America have experienced a gradual modification of lifestyle over several centuries, where a diet based on high intake of carbohydrate replaced the traditional hunter-gatherer diet with high protein intake. Similar changes have occurred in third world countries, but in many cases the transition has taken place over decades – not centuries. A combination of increased access to food and decreasing demands with respect to physical activity has led to increasing prevalence of obesity, dyslipidaemia, hypertension and hyperglycaemia/diabetes, a combination of features often referred to as the metabolic syndrome."

By 2025, three out of four people with diabetes will be living in third world countries, and similar trends are likely for the other components of the syndrome. Simpler and low-cost preventive measures are urgently needed to take place everywhere [9]. A study planned and conducted in China showed that lifestyle modifications, like physical activity and healthier diet, were effective in preventing diabetes [10]. The study was subsequently repeated in Finland, USA, Canada and India, and all these studies confirmed the original results. Based on the experiences from China and India it ought to be possible to develop prevention programmes and strategies that are not only effective, but also locally acceptable and feasible [9].

*D&MS *targets a readership comprising clinicians, scientists, paramedical personnel, nutritionists, and health care personnel working in the field of diabetes. Original research work and reviews of interest to the above group of readers will be considered for publication in the journal.

The Brazilian Diabetes Society is intended to be a nationally and internationally recognized as a reference institution and diabetes knowledge centre leaning on ethical, medical, social, and transparency principles independent of any personal, financial, and corporate interests. The SBD comprises health care professionals from all over Brazil and abroad in large numbers.

As an open access journal, *D&MS *not only gives worldwide visibility to SBD and its activities but also offer a natural place for publishing the proceedings and abstracts of the SBD's scientific meetings as supplements. In this way, the journal can act as a communication forum for issues of importance to the relevant community, far beyond the SBD's current membership, and can potentially attract further members. In short, having an open access journal is an excellent way for a professional society like SBD to fulfil its mission of furthering knowledge in its field.
